# Synthesis of MgB_2_ at Low Temperature and Autogenous Pressure

**DOI:** 10.3390/ma7053901

**Published:** 2014-05-15

**Authors:** Ian D. R. Mackinnon, Abigail Winnett, Jose A. Alarco, Peter C. Talbot

**Affiliations:** 1 Institute for Future Environments, Queensland University of Technology, Brisbane, QLD 4001, Australia; E-Mails: a.winnett@qut.edu.au (A.W.); jose.alarco@qut.edu.au (J.A.A.); p.talbot@qut.edu.au (P.C.T.); 2 Science and Engineering Faculty, Queensland University of Technology, Brisbane, QLD 4001, Australia

**Keywords:** magnesium diboride synthesis, sodium borohydride, superconductivity

## Abstract

High quality, micron-sized interpenetrating grains of MgB_2_, with high density, are produced at low temperatures (~420 °C < *T* < ~500 °C) under autogenous pressure by pre-mixing Mg powder and NaBH_4_ and heating in an Inconel 601 alloy reactor for 5–15 h. Optimum production of MgB_2_, with yields greater than 75%, occurs for autogenous pressure in the range 1.0 MPa to 2.0 MPa, with the reactor at ~500 °C. Autogenous pressure is induced by the decomposition of NaBH_4_ in the presence of Mg and/or other Mg-based compounds. The morphology, transition temperature and magnetic properties of MgB_2_ are dependent on the heating regime. Significant improvement in physical properties accrues when the reactor temperature is held at 250 °C for >20 min prior to a hold at 500 °C.

## 1. Introduction

Since the determination of a superconducting transition temperature [1] for MgB_2_, a wide range of synthesis methods has been employed to produce powders and single crystals. These methods include solid state [2–4], liquid and/or gas phase reaction with pressure [5,6] and novel combustion syntheses [7,8]. While powder-in-tube methods [9,10] are developed for wire production, more extensive utilisation of MgB_2_ is likely to occur with development of facile methods for bulk synthesis. MgB_2_ synthesis methods include mixing elemental powders sintered at high temperature (~900 °C) [2,11], combustion synthesis at >1200 °C [12], mechano-chemical mixing followed by sintering [3] at >600 °C or with pressure and high temperature (>20 kbar and 1400 °C) [6].

A comprehensive review by Carenco *et al.* [13] describes many synthesis methods for nano-scale metal borides including studies on autogenous pressure reactions. Solid-state autogenous syntheses are predominantly directed at hexaborides while liquid phase syntheses using solid precursors under autogenous pressure have been used to produce nano-scale diborides such as NbB_2_ and TiB_2_ [13]. In other cases, liquid phase precursors such as TiCl_4_ are combined with a solid precursor such as NaBH_4_ in an autoclave to produce nanoparticles of metal diboride [13]. In many of these studies, the objective is production of nano-scale crystalline borides of high purity which, with liquid phase synthesis, may be questionable [13]. Nevertheless, while autogenous pressure reactions (e.g., using an autoclave) show promising results, there is a paucity of data on the actual pressure(s) under which these solid state or liquid autogenous pressure reactions occur.

For many syntheses, the reaction depends on the formation of Mg liquid and/or gas at high temperature or may not afford bulk manufacturing at industrial scale [13]. Synthesis of bulk MgB_2_ as dense powders without the use of high pressure or the powder-in-sealed-tube (PIST) method [9] is also a challenge [14] for industrial applications. Zeng *et al.* [14] demonstrate significant improvement in powder density using a direct diffusion synthesis at 850 °C. This method results in an improvement of *J*_c_ across a range of physical conditions although synthesis requires high temperature conditions [14] similar to that currently used for powder-in-tube [9].

In general, lower temperature synthesis or solution-based methods are likely to result in products with potential for higher density and commercial production of this technologically important material. Recently, Portehault *et al.* [15] demonstrated a generic solution process for synthesis of borides including NbB_2_ and HfB_2_, which form at lower temperature (~400 °C–900 °C) utilising inorganic molten salts. The optimum temperature for NbB_2_ formation using a LiCl/KCl melt is relatively high at 900 °C albeit with the benefit of high purity and nanometre sized crystallites [15].

The use of autogenous pressure is implicit in the production of wires *via* the PIST method [9] and is also demonstrated for the production of MgB_2_ powders. For example, a synthesis along similar lines to that of Portehault *et al.* [15], is used by Lu *et al.* [16] to form MgB_2_ between 350 °C and 450 °C. In this work [16], MgH_2_ and LiBH_4_ are milled with minor amounts of TiCl_3_ in a sealed, oxygen-free environment and heated to 400 °C–450 °C for twelve hours. The reaction produces nanometre-sized particles with typical magnetisation and structural characteristics for MgB_2_ [16]. In another example, Pol *et al.* [17] rapidly heated to 750 °C elemental Mg and B in a nitrogen atmosphere to produce MgB_2_ of high quality with ~85% yield. More recently, Chen *et al.* [18] have demonstrated production of single crystal MgB_2_ using a hybrid physico-chemical vapor deposition (HPCVD) method at constant pressure of 21kPa and ~700 °C.

Autogenous pressure offers the potential to operate at lower temperature compared to conventional solid state synthesis methods with control determined by reaction time and absence of reactive species such as oxygen. In this work, we describe an autogenous reaction without the use of an additional dehydrogenation agent to produce MgB_2_ between 420 °C and 500 °C using NaBH_4_ and Mg metal. High yields of dense, micrometer-scale MgB_2_ powders are obtained through control of the reaction temperature and heating rate.

## 2. Results and Discussion

### 2.1. Reaction Protocols

**Figure 1.**
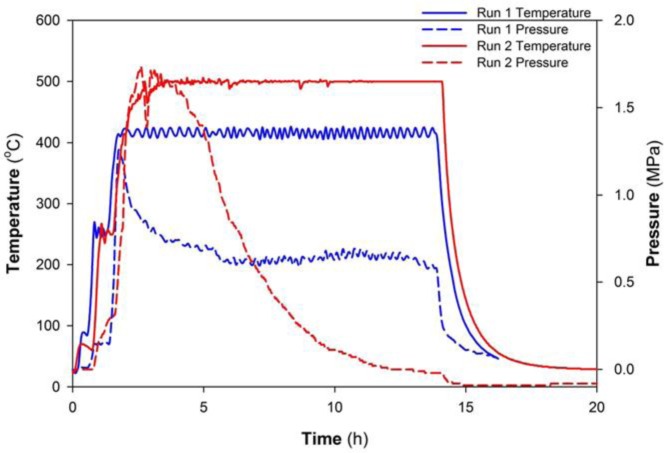
Temperature and pressure profiles for Runs 1 and 2.

A summary of reaction parameters for each run that generated MgB_2_ is shown in Table 1. Some runs are repeated for validation of reaction products. Peak temperatures measured at the thermocouple for these reactions ranged from 420 °C to 500 °C with varying yield of MgB_2._ In all cases, MgB_2_ is the dominant phase. For Runs 1 and 2, the same heating profile is used up to the target temperature as exemplified in Figure 1. That is, a heating rate at 10 °C/min with a 20 min hold (*i.e.*, zero ramp) at 50 °C and 250 °C, respectively. For Run 3, the ramp rate is 10 °C/min with a 20 min hold at 50 °C and, subsequently, a 5 h hold at 250 °C. For Run 4, the same temperature increase of 10 °C/min is maintained, but without a hold until 500 °C.

Similar parameters for reactions that did not generate MgB_2_ as the predominant phase using the same starting materials are shown in Table 2. Peak temperatures for these reactions ranged from 200 °C to 400 °C and follows similar heating profiles to Runs 1−2 with the exception of Run 6 which is without a hold until 400 °C.

In general, the reactor shows distinct layering of products from the base of the vessel, around which the heating element is arranged, to the top region which contains the release valves for gas discharge. This layering reflects a variation in temperature profile with the top of the reactor colder than the centre of the reactor where the thermocouple is situated. In general, comparison of set points, controller values and thermocouple values suggest that the variation in temperature from the centre of the reactor to its extreme is approximately 10%–15% relative. For the experiments listed in Table 1, phases such as NaH and NaBH_4_ occur at the top of the reactor and are readily separable from the primary MgB_2_ product, sometimes with residual Mg, occurring at the bottom of the reactor.

Results from these experiments imply the formation of gaseous phases at temperatures higher than that nominally specified by the *in situ* thermocouple. In order to evaluate the temperature profile within the reactor—to a rough approximation—“blank” experiments with no reagents or only Mg powder are also undertaken. These experiments suggest that under specific heating rate regimes, the bottom of the reactor may achieve temperatures for short periods of time significantly higher (up to 150 °C) than the value recorded by the thermocouple located in the centre of the reactor. For example, an Mg strip placed on the bottom of the reactor and subject to conditions equivalent to Run 4 results in a sharp increase in pressure to 0.25 MPa with subsequent reduction in pressure similar to Runs 1–4 over a ten hour reaction period. This result and observations of the heated Mg strip after reaction (images not shown), suggest that the bottom of the reactor experiences temperatures > 600 °C for short periods.

### 2.2. Formation of MgB_2_

**Figure 2.**
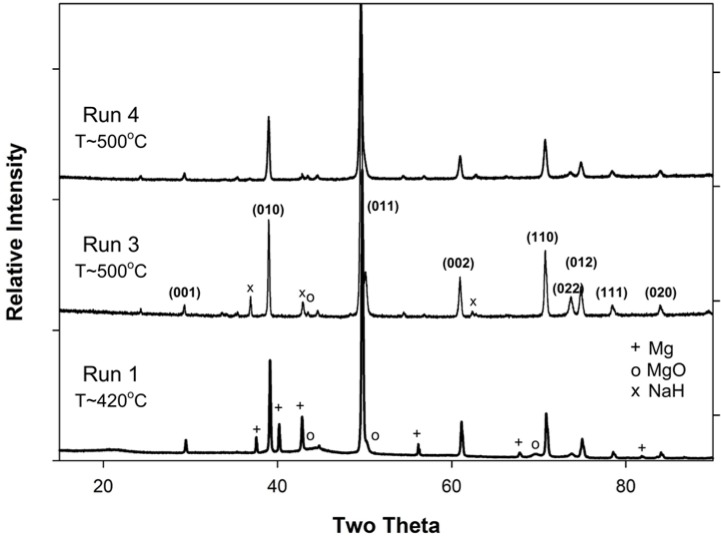
XRD patterns for Runs 1, 3 and 4; indices are for MgB_2_.

Figure 1 shows the temperature and pressure profiles for reactions listed in Table 1 as Runs 1 and 2. A characteristic reduction in pressure within the reactor occurs after a peak value at or near 500 °C (e.g., Run 2). In both cases, MgB_2_ is formed as determined by X-ray diffraction (XRD) patterns (see Figure 2; data not shown for Run 2). The lower temperature synthesis at 420 °C (Run 1) also shows the presence of minor Mg which suggests the reaction to form MgB_2_ is incomplete. A minor amount of MgO is also present in the XRD pattern for Run 2. Semi-quantitative XRD analyses and weight of products from a typical reaction (e.g., Run 2) indicates an MgB_2_ yield of ~75% on the basis of Mg input.

**Figure 3.**
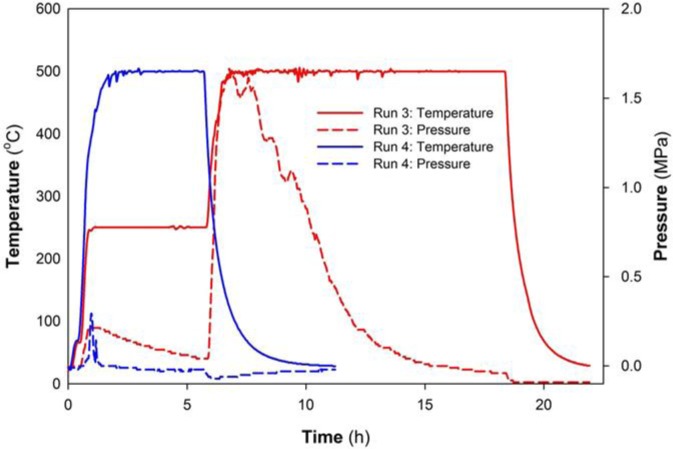
Temperature and pressure profiles for Runs 3 and 4.

Figure 3 shows the temperature and pressure profiles for Runs 3 and 4. This plot compares the longer hold period for 5h at 250 °C with a direct heating to 500 °C. A reduction in pressure occurs not only during the 250 °C hold but also over the 500 °C heating range for Run 3. In this case, an XRD pattern of material at the bottom of the reactor shows MgB_2_ is predominant (>96%) with minor amounts of NaH and MgO (Figure 2; Run 3).

Visual inspection of the top of the reactor indicates a grey or dull white coloured material which is consistent with NaH. In some regions, metallic coloured material (which spontaneously ignites on contact with water) is consistent with the presence of Na and/or NaH. XRD of material from the top of the reactor shows that NaH is the predominant phase. Figure 3 also shows that for Run 4 maintaining a heating rate of 10 °C/min until the reactor temperature is 500 °C causes a rise in pressure, but to a significantly lower value than in Runs 1–3. The maximum pressure achieved during Run 4 is 0.3 MPa for a limited time (approx. 30 min) after which a very rapid drop in pressure occurs while the temperature remains at 500 °C.

**Figure 4.**
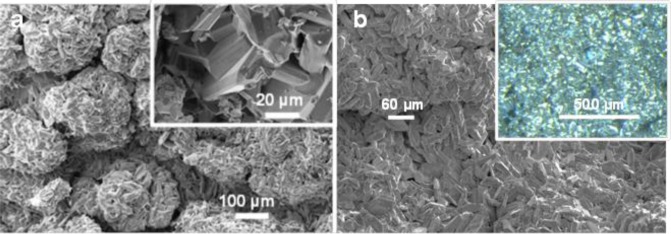
SEM images of MgB_2_ from (**a**) Run 2 shows pseudomorph aggregates of Mg grains. Higher resolution SEM image (inset) shows euhedral angular features of grains and (**b**) Run 3 shows greater extent of inter-penetrating grains and optical micrograph (inset) with multiple orientations of MgB_2_ surfaces.

Figure 4a shows SEM images of product from Run 2 in which well-formed intergrown MgB_2_ grains are clustered within aggregates approximating the size (>100 μm to ~200 μm) of the original Mg grains (e.g., pseudomorphs) used as reactants. Higher magnification images (inset Figure 4a) show euhedral platy morphology with hexagonal shaped and bi-pyramidal or bi-prismatic interpenetrating forms with fine lamellar striations on well-defined surfaces. Individual MgB_2_ grains range in size from 20 μm to 60 μm in maximum dimension and 5 μm to 10 μm in minimum dimension.

Figure 4b shows a typical SEM image of the reaction product from Run 3 in which dense growths of fine-grained, euhedral shapes with inter-grown features are also evident. Reflecting surfaces of MgB_2_ are highlighted in the optical image (inset to Figure 4b) and indicate that these surfaces are uniformly shaped but in many different orientations. In incident light, these surfaces have a gold colour. In general, these MgB_2_ grains range from 40 μm to 80 μm in maximum dimension and between 10 μm and 20 μm in minimum dimension.

**Figure 5.**
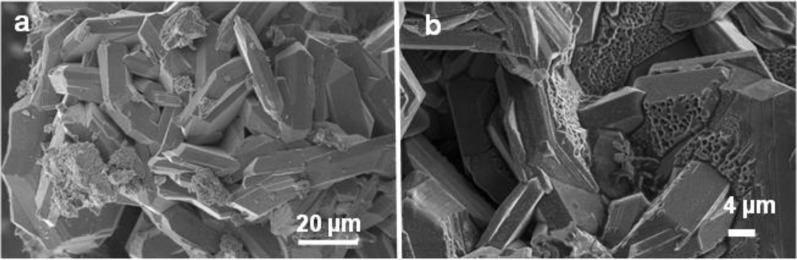
SEM images of MgB_2_ formed during Run 3 at 500 °C (see Table 1). Note the presence of vesicles on some surfaces suggesting vapor phase reaction.

SEM images of MgB_2_ from Run 3 in Figure 5a show euhedral, interpenetrating growth of densely packed grains. Figure 5b shows, at higher magnification, the presence of circular features on exposed surfaces that suggest formation of vesicles or gas phase interaction. EDS of these regions indicates a predominantly Mg and B composition.

**Figure 6.**
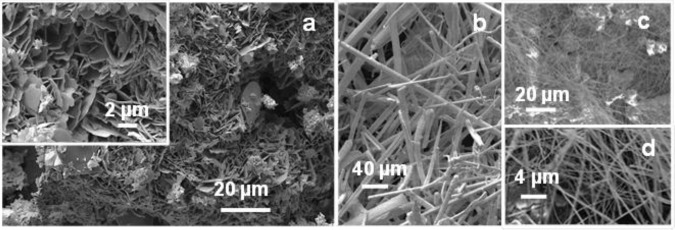
SEM images from (**a**) Run 4 at 500 °C showing MgB_2_ as lower density, irregular grains compared with Run 3; (**b**) NaH obtained from the top of the reactor after Run 4; (**c**) and (**d**) Filamentous material from Run 7 showing MgH_2_.

Figure 6 shows SEM images of typical morphologies from other reactions such as Run 4 (Figure 6a,b) and Run 7 (Figure 6c,d). For Run 4, the XRD pattern shown in Figure 2 indicates that MgB_2_ is the dominant phase at the base of the reactor. However, the morphology and density of MgB_2_ grains shown in Figure 6a is in stark contrast to that formed in Runs 2 and 3. The morphology is anhedral, thin disc-like and similar to a “cornflake” texture with minimum dimension < 1 μm and maximum dimension 5 μm to 10 μm. Figure 6b shows typical fibrous or needle-like morphology for NaH which occurs at the top of the reactor chamber. XRD data from this sample confirms the presence of NaH with minor amounts of NaBH_4_.

### 2.3. Low Temperature Reactions

A series of reactions were also performed at lower temperature with similar heating profiles as listed in Table 2. In all cases, the degree of autogenous pressure developed during the reaction is significantly lower than reactions listed in Table 1 where the peak temperature is higher. The highest pressures achieved are 0.36 MPa and 0.61 MPa for the reactions at 400 °C (Runs 5 and 6), while all other reactions recorded pressures in the low kPa range. For both Runs 5 and 6, minor amounts of MgB_2_ are detected in the products of reaction along with Mg, NaBH_4_ and NaH.

Figure 6c,d show SEM images of material collected at the top of the reactor after Run 7. This experiment held temperature at 300 °C for two and a half hours and then cooled to room temperature. EDS and XRD analyses show that this material is MgH_2_. The fibrous nature of this material is consistent with observations by Zhu *et al.* [19] on the formation of MgH_2_ under various conditions of hydrogen pressure.

XRD data from the runs shown in Table 2 are dominated by the starting materials particularly for Runs 7–9. Runs 8 and 9 evaluate the influence of mixing in the agate mortar with Run 8 an example for which the mixed reactants generate a moderate level of pressure (~50 kPa). However, for Run 9 in which reactants are not mixed prior to the reaction, generation of significant pressure in the reactor with increase in temperature did not occur.

### 2.4. Physical Properties of MgB_2_

Surface area analyses of MgB_2_ from Runs 2–4, all to 500 °C, are consistent with observations from SEM images. The sample from Run 4 is sufficiently porous to register a surface area value of 2.88 m^2^/g with a total pore volume of 0.005 cm^3^/g. Samples from Runs 2 and 3 did not register a surface area value using N_2_ as the absorbent.

**Figure 7.**
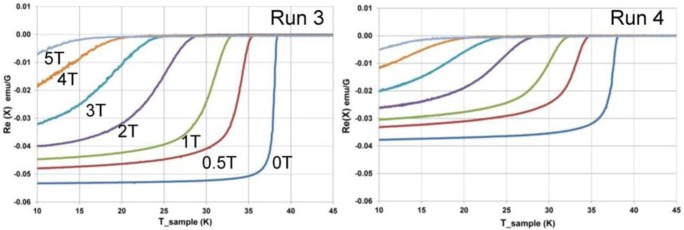
AC magnetic susceptibility for MgB_2_ formed from Runs 3 and 4 showing variation with applied magnetic field. At zero field *T*_c_ = 38.5 K for Run 3 and *T*_c_ = 38.0 K for Run 4. Vertical axes are the same scale. Note difference in AC susceptibility for Run 4 compared with Run 3.

Measured AC magnetic susceptibility data for MgB_2_ from Runs 3 and 4 are shown in Figure 7. For zero applied field, the superconducting transition temperature for Run 3 at 38.5 K is marginally higher than that determined for MgB_2_ from Run 4 at 38.0 K. With variation in applied magnetic field, both runs show a similar decrease of the transition temperature with increasing field strength. Figure 7 also shows that the sample from Run 3 has a higher absolute value for susceptibility compared with the sample from Run 4 over the same temperature range.

### 2.5. Autogenous Pressure

Sodium tetrahydroborate, or sodium borohydride (*i.e.*, NaBH_4_), is widely used in laboratory and industrial practices [20,21] and is now under extensive re-evaluation in conjunction with other hydrogen bearing compounds (e.g., MgH_2_, Mg_2_FeH_6_, Mg_2_NiH_4_) due to strong interest in industrial applications for hydrogen storage [22]. Research on borohydrides as solid state hydrogen storage materials are well documented in the literature [23] which predominantly focuses on decomposition reactions and dehydrogenation rates over a range of temperature and pressure conditions [20,24,25]. As noted by Martelli *et al.* [20], the temperature at which hydrogen is released from a compound is strongly influenced by the applied pressure and heating rate. In addition, related work shows that the hydrogen release rate in borohydrides is affected by the presence of boron [24] and/or Mg [23,25–27].

Both *in situ* syntheses using autogenous pressure [9,17], as well as earlier work by Prikhna *et al.* [28], use Mg powder and amorphous boron as starting materials. Temperatures greater than 650 °C—the melting point for Mg at 0.1 MPa—are used in order to ensure Mg liquid is in close contact with reactive boron and that any Mg is not lost through evaporation or oxidation [9,17]. Optimum reaction rates and yields by this method occur at 750 °C or higher and produce nanometre-sized MgB_2_ grains [17]. These processes require the mixed reactants to be densely packed within a sealed vessel [17] or compressed into a stainless steel tube at ~1 GPa using a hydraulic press [9].

In comparison in this work, the reactants, as a manually mixed paste, occupy less than one third the volume of the reactor. The temperature-pressure data and resulting products from these reactions suggest that autogenous pressure in Runs 1–7 is derived from the production of gas(es) during the reaction(s). The nature of the gas(es) present in the reactor over the temperature range is inferred to include hydrogen, sodium, diborane and magnesium. In addition, the temperature-pressure profiles (e.g., Figures 1 and 3) consistently show a significant increase in pressure as the temperature increases after the 250 °C hold. The presence of minor MgO in some products also suggests that very small amounts of residual water remain within the reactor connections even after thorough cleaning. We shall examine the general attributes of phase decomposition and gas evolution during these reactions in the sections below.

#### 2.5.1. Key Reactions

Over the temperature range 25 °C to 500 °C, a number of key reactions are evident from the composition of the major products formed. The precise mechanism(s) and type(s) of reactions are difficult to detail without additional data collected during the reactions (e.g. composition of gases generated at specific temperatures). However, reactions that reflect the most likely, or dominant, transformations are presented in this section. These key reactions involve decomposition of NaBH_4_ reactant to form H_2_ and/or B_2_H_6_ gases, at relatively low temperatures (<250 °C), accelerated decomposition of NaBH_4_ in the presence of Mg along with formation of intermediate phases such as MgH_2_ at moderate pressure (<2 MPa), decomposition of MgH_2_ and NaBH_4_ to form reactive Mg and Na and possibly Mg(BH_4_)_2_ at higher temperatures (200 °C< *T* <400 °C) and formation of MgB_2_ and/or residual gas(es) between 400 °C and 500 °C. Formation of NaH along with unreacted or residual NaBH_4_ occurs between 300 °C and 450 °C on the upper section of the reactor given sufficient production of H^−^. The overall reaction for this system can be summarised as follows:

$$2{\text{NaBH}}_{4}+\text{Mg}\to 2\text{Na}+{\text{MgB}}_{2}+4H_{2}(g)$$ (1)

However, other intermediate reactions are evident from the analysis of all reaction products including those undertaken at lower temperatures (see Tables 1 and 2).

A significant increase in pressure within the reactor occurs between 250 °C and 500 °C. This pressure increase is consistent with advanced decomposition of NaBH_4_ in the presence of Mg or MgH_2_ [24,27] and, most likely, exothermic intermediate reactions over this temperature range [13]. According to Zhu *et al.* [19], MgH_2_ forms at 400 °C using Mg(s) and H_2_(g) with up to 4 MPa hydrogen pressure. At lower pressure up to 1 MPa, Mg and MgH_2_ occur at this temperature [19]. While formation of MgH_2_ from Mg powder via gas-solid reaction at low temperatures is diffusion limited [29], high quality fibres can form via high pressure reaction with Mg and H_2_(g) [19]. Figure 4 in Zhu *et al.* [19] suggests that this gas-solid reaction occurs across a wide temperature range from <300 °C to 500 °C with higher quality MgH_2_ at higher pressure. Data from Run 6, for the reaction held at 300 °C shows that MgH_2_ is formed and suggests that this phase is an important intermediate in the overall reaction to form coarse grained MgB_2_. This experiment (Run 6) also implies that H_2_ is present as a gas above 250 °C and ~0.07 MPa pressure.

The reaction parameters for Run 3, for which a five hour hold is maintained at 250 °C, indicate that a gas is consumed at this temperature due to the gradual reduction in reactor pressure (Figure 3). We have established above that H_2_ is present as a gas over a range of temperatures in this type of reaction. In addition, Zhu *et al.* [19] and others [26] have shown that hydriding chemical vapour deposition is a viable mechanism for the production of MgH_2_ between 250 °C and 600 °C and 1 MPa to 4 MPa pressure. Thus, we propose that the reduction in pressure for Run 3 is predominantly a result of the formation of MgH_2_. The step-like nature of the pressure drop at constant temperature may be due to competing reactions that include not only the production of MgH_2_ but also its decomposition in a composite mix of NaBH_4_ and MgH_2_ (see below).

A rapid increase in temperature that follows the hold at 250 °C is likely to generate localised heating of Mg powder at the bottom of the reactor to more than 500 °C due to a finite delay response time between the thermocouple and temperature controller. Between 600 °C and 650 °C, the vapour pressure for Mg is between 0.13 kPa and 0.41 kPa and thus, may contribute to an increase in pressure as temperature is raised.

Mg(BH_4_)_2_ decomposes to form MgH_2_ between 320 °C and 400 °C [28] although a lower temperature 290 °C is also noted [30]. Mg(BH_4_)_2_ is not detected in the products listed in Tables 1 and 2. Zhang *et al.* [31] suggest that it will form in a gas-solid reaction with B_2_H_6_ and solid MgH_2_ at temperatures up to 220 °C. Thus, this reaction may occur as the reactor ramps up in temperature to 250 °C, but direct evidence is not available from the data collected in these experiments.

#### 2.5.2. NaBH_4_ Decomposition

Decomposition of NaBH_4_ is an important component of this reaction. A computational study by Cakir *et al.* [32] provides insight into the decomposition process under conditions outlined in this work (~400 °C). Mass transport within a solid crystal occurs *via* lattice defects which, as noted by Cakir *et al.* [32], occur predominantly in NaBH_4_ as charged defects of Na^+^ and BH_4_^−^ ions. Density functional theory calculations show that the formation energies of vacancies corresponding to these ions are the lowest of all possible species and thus, are the primary means for transport of B, H and Na in bulk NaBH_4_ [32]. According to this model, substitution of the BH_4_^−^ ion with H^−^ also readily occurs and results in BH_3_ vacancies which are not easily incorporated into the lattice. The decomposition of BH_4_^−^ to H^−^ and BH_3_ occurs at the surface of NaBH_4_ with H^−^ ions locally forming NaH within the lattice [32]. Thus, formation of BH_3_ gas which readily polymerizes to form B_2_H_6_ and higher forms [33] is promoted under these conditions.

Computational and experimental studies confirm that the decomposition of NaBH_4_ is enhanced in the presence of Mg [27] or MgH_2_ [33] or MgB_2_ [32] and may commence at temperatures up to 100 °C lower than without Mg [27]. The melting point for NaBH_4_ is 505 °C at standard pressure [25] and is also shown to decrease in the presence of other metals including Mg [20,23] but may increase to 535 °C under 0.1 MPa hydrogen pressure [20]. Thus, dehydrogenation or decomposition of NaBH_4_ may commence at temperatures much lower than 400 °C in the presence of Mg while the melting point may increase due to the imposition of autogenous hydrogen pressure [20]. This condition will extend the potential to generate BH_3_ from the surface of NaBH_4_ over a wider temperature range and, consequentially, production of H_2_.

In a study of a composite mix of NaBH_4_ + MgH_2_, Kato *et al.* [34] shows that the decomposition of MgH_2_ commences at ~230 °C and is complete at ~330 °C. In addition, the decomposition of NaBH_4_ in this composite mix begins at ~250 °C and is induced by the migration of Mg to the reaction front [34]. Thus, if MgH_2_ is formed at lower temperature in Runs 1–6, the decomposition of both MgH_2_ and NaBH_4_—if in intimate mix—occurs at significantly lower temperature than 400 °C. As noted by Kato *et al.* [34], this suggests that reaction (2) is also an important process.

$$2{\text{NaBH}}_{4}+\text{Mg}\to {\text{MgB}}_{2}+2\text{NaH}+3H_{2}(g)$$ (2)

#### 2.5.3. Other Reaction Parameters

While the generated morphologies and migration of products to the reactor lid, for example, are evidence of vapour phase transfer, another important indicator that gas phase reactions are involved in Runs 1–6 is the reduction of pressure in the reactor while at constant temperature. In all runs, and as shown in Figures 1 and 3, the reduction in pressure to a vacuum or ambient occurs prior to the reaction achieving ambient temperature. This trend suggests that for experiments held at 500 °C, the primary formation mechanism for MgB_2_ involves gas phase reaction. Data for experiments at lower temperature (>250 °C; Runs 1, 5 and 6) also suggest that gas phase reaction is a mechanism, perhaps of lesser influence, at these temperatures. This implication arises from the temperature-pressure profile during the experiment as well as the presence of MgH_2_ in Run 6. The precise identity of these gases at specific temperature settings is not available as it is difficult to sample from within the reactor while maintaining pressure. However, we infer from previous studies in similar systems that these gases are likely to be B_2_H_6_, H_2_, Na and possibly Mg [31,32,35].

Decomposition of NaBH_4_ occurs at low temperature when intimately mixed with a metal chloride and heated above 50 °C [31]. Evolution of B_2_H_6_ is optimal at 90 °C while production of H_2_ increases with higher temperature to ~110 °C [31]. In the absence of metal chloride, B_2_H_6_, which may form from the slow decomposition of NaBH_4_ (see above; [32]), generates hydrogen with heating. The presence of B_2_H_6_ and its polymer variants will depend on pyrolysis temperature as shown by Bragg *et al.* [35] who demonstrate that B_2_H_6_ generates H_2_ above 80 °C. Hydrogen generation from heated B_2_H_6_ is characterised by a short-lived period of rapid production [35] and results in the formation of intermediate boron hydride species with increase in temperature [35,36]. At higher temperatures above 180 °C, these intermediate compounds condense to form a yellow solid [35,37]. While this solid is not identified in earlier work, it is reasonable to suggest the material is a higher borohydride such as B_5_H_9_ [37].

This earlier work suggests that at low temperatures, evolution of hydrogen and borohydride gas is possible and is partially responsible for the increase in pressure measured in these reactions up to 250 °C. This increase in pressure is enhanced by holds at 50 °C and 250 °C which ensure continued decomposition of NaBH_4_ (in the presence of Mg or MgH_2_) and production of H_2_. At this time, a boron-rich solid may condense in regions that are slightly lower in temperature within the reactor. With increased temperature, the boron-rich solid may also become unstable or susceptible to formation of MgB_2_ as noted above.

The melting point of Na at 0.1 MPa [38] is 97.7 °C and the vapour pressure at 325 °C is 5.6 Pa (or 0.005 kPa), while the vapour pressure at 420 °C and 0.1 MPa is 0.089 kPa [38]. If Na is formed via reaction (1) above, these parameters suggest that for Runs 1–6, the presence of liquid and/or gaseous Na is likely.

### 2.6. Physical Properties of MgB_2_

Images via optical and electron optical methods (Figures 4 and 6) show that grain sizes of MgB_2_ are micrometre-scale and, in both cases for which the heating rate is held at 250 °C, an intimate mixing and interpenetration of grains is apparent (Figures 4 and 5). In general, these MgB_2_ grains range in size from 40 μm to 80 μm in longest dimension and show well defined crystal faces with foreshortened dimension orthogonal to the basal plane. The grain size of MgB_2_ heated directly to 500 °C (without a hold at 250 °C) is generally smaller (~2 μm to 5 μm) and while intermixed, does not show interpenetration of hexagonal-shaped grains at the edges or faces of other individual grains. Images in Figures 4 and 5 suggest a higher density of grains are produced by heating rates that include a hold at 250 °C compared with direct heating to 500 °C. The BET surface area values for samples from Run 4 compared with undetectable values for Runs 2 and 3 confirm this interpretation.

Direct vapour phase production [17] of MgB_2_ in a nitrogen atmosphere at 750 °C produces nanometre-scale material ranging in size from 40 nm to 80 nm with a BET surface area of 13.9 m^2^/g and a pore diameter of 0.96 cc/g. The HPCVD method developed by Chen *et al.* [18] generates well-formed, euhedral MgB_2_ crystals ranging in size from 10 μm to 30 μm. The crystal shapes identified by Chen *et al.* [18] show a clear evolution of growth stages to form hexagonal microprisms including transition through spherical icosahedron and hexagonal icosahedron stages. This type of evolution is not observed with products from Runs 1–4 and implies a different reaction mechanism for the process in this work. While the process used by Chen *et al.* [18] is a direct vapour phase condensation—based on experimental conditions and results achieved—this is unlikely to be the process for Runs 1–4 in this work. The presence of gas(es) in the reactor is clear but the pressure profile(s) and temperature settings suggest that gas-solid and gas-liquid reactions are evident.

AC magnetic susceptibility data for all reactions that produced MgB_2_ show typical temperature dependence curves for a superconducting transition at *T*~38K. However, detailed examination of magnetic susceptibility for samples from Runs 3 and 4 shows that subtle differences in the quality of physical properties can be related to the method of formation. For Run 4, where the reactor heating does not include a hold until 500 °C, a higher porosity and lower density of grains (see Figure 6a) manifests as a marginally lower value for *T*_c_ and a lower absolute value for total AC susceptibility. In addition, the quality of product and capacity to maintain superconductivity and large magnetic moment with an applied magnetic field is also influenced by this difference in grain quality.

**Figure 8.**
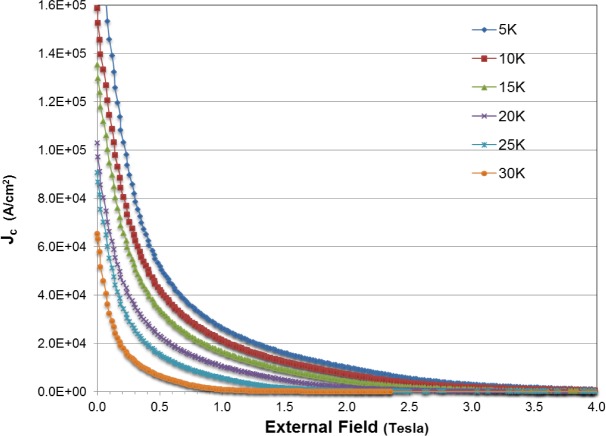
Critical current density (*J*_c_) for MgB_2_ sample from Run 3 as a function of external field over the temperature range 5 K to 30 K.

Magnetic hysteresis curves (not shown) determined using Vibrating Sample Magnetometry (VSM) also reflect a difference in grain quality. For example, first critical values (*H*_c1_) determined from the curves at 20 K for the Run 3 sample is 79 mT while for the Run 4 sample *H*_c1_ is 26 mT (after allowing for relative weights of samples). Similar differentials in values for *H*_c1_ occur between these samples for the temperature range 5 K to 30 K. These values suggest that grain connectivity for the sample from Run 3 is significantly better than for Run 4. Figure 8 shows the calculated critical current density (*J*_c_) determined by the well-known Bean model [39] for the sample from Run 3. For this calculation, the average grain diameter determined experimentally is used to estimate the diameter of the cylinder for the Bean model. These values for *J*_c_ are comparable to other measures of MgB_2_ [40,41] and show the quality of product from an autogenous pressure reaction. A more detailed analysis of physical properties for an extended suite of MgB_2_ samples produced by these methods is in preparation.

## 3. Experimental Section

### 3.1. Synthesis

Molar ratios of coarse magnesium powder (0.656 g; <50 mesh size; 99.9% purity) and sodium borohydride powder (2.046 g; 99.99% purity) supplied by Sigma-Aldrich (Castle Hill, Australia) are weighed, mixed and placed into a 50 mL Parr reactor within a controlled atmosphere glove box containing Argon (99.99%). Starting materials are pre-heated in an evacuated chamber adjacent to the glove box in order to reduce to the dehydrated state and then transferred to an inert atmosphere. Water and oxygen content in the glove box is normally less than 1 ppm. After weighing, the starting materials are manually mixed in an agate mortar until a consistent texture and greyish colour are achieved. Minor amounts of sample lost during this process are estimated at less than 1% of total volume.

The Parr reactor is designed with an internal fixed head and cylinder of Inconel 601 steel with a graphite seal to accommodate a maximum pressure of 20 MPa and a maximum average temperature of 500 °C. Before each reaction, the cylinder is thoroughly cleaned with dilute acid, washed with deionised water and dried in the pre-heating chamber up to 120 °C. The lid is rinsed with water and dried with a hair dryer. In principle, this process removes all products from a prior run and minimises (but does not eliminate) traces of residual water.

The reaction mixture is added to the Parr reactor, sealed tightly and removed from the glove box. The reaction chamber is heated according to a standard protocol via thermocouple controller. The change in pressure is monitored during the reaction using a dial pressure gauge and an Ashcroft transducer mounted atop the reaction chamber. The temperature sensor is centred within the reactor and both temperature and pressure are recorded every second. The temperature sensor is not embedded within the precursor materials which, at the start of synthesis, are located at the bottom of the chamber. The typical pressure-temperature profiles shown in Figures 1 and 3 use data captured at 100 s intervals.

On cooling the reaction chamber to room temperature, the reactor is opened in the argon-filled glove box *via* slow pressure equilibration using a gas release valve. Degradation of these samples occurs after periods of more than 24 h exposure in air as noted by Zhu *et al.* [11]. In general, material removed from the reaction chamber is placed immediately on substrates or contained within controlled atmosphere environments for subsequent characterisation. An exception to this is the reaction at 420 °C shown in Figure 1, for which the reactor was opened in air and samples were washed with deionised water.

A consistent heating rate of 10 °C/min is used in all reactions albeit at different temperatures, the heating rate is held constant for varying periods of time. In general, the reactor heating rate is held constant at: (i) ~50 °C; (ii) 250 °C; and (iii) target temperature (usually 500 °C) for variable periods of time. These specific heating rates and constant temperature periods are identified in Tables 1 and 2. High yields of dense MgB_2_ powders are obtained through control of the reaction temperature and heating rate.

### 3.2. Characterisation

Reaction products are characterized by optical and electron-optical methods and X-ray diffraction. A Leica multi-focus, stereo optical microscope and a Zeiss Sigma variable pressure Field Emission SEM with Oxford Instruments silicon drift detector (SDD) (Carl Zeiss Pty Ltd., North Ryde, Australia) are used for microscopy observations and energy dispersive spectroscopy (EDS) elemental analysis. Samples are prepared for SEM/EDS by placing a thin layer of powder onto aluminium stubs with double sided carbon tape. In general, samples are not coated with a conductive coating to avoid analytical interference(s). Elemental analysis is carried out at an accelerating voltage of 20 kV at 8.5 mm working distance. Excessively charging samples are imaged at lower accelerating voltages of 5 kV or 10 kV. If charging is excessive when the sample is uncoated, some samples are re-examined after deposition of a gold coating.

X-ray diffraction data are collected via a PANalytical X’Pert MPD X-ray diffractometer (Spectris Australia Pty Ltd., Sydney, Australia) using Co Kα radiation. Data are collected for 4° < 2θ < 90° at a step size of 0.02° 2θ on samples as received (*i.e.*, not crushed). Depending on the particle size of the reaction products, X-ray data are collected with a parallel beam configuration, rather than Bragg-Brentano geometry typically used for finely ground powders. Surface area determinations using the BET method and N_2_ gas are obtained with a Micromeritics Tristar II instrument (Particle and Surface Sciences Co., Gosford, Australia). Magnetic susceptibility is measured on bulk powders using a Cryogenic Ltd Mini Cryogen-free System (Cryogenic Ltd., London, UK) with a 5 T magnet and integrated variable temperature insert which allows for temperature control at the sample between ~300 K and 1.5 K. Hysteresis is measured in the VSM mode at a frequency of 20.4 Hz using a time constant of 300 ms.

## 4. Conclusions

Formation of MgB_2_ is achievable at moderate temperatures (~420 °C < *T* < ~500 °C) using pre-mixed starting materials of Mg powder and NaBH_4_ within an Inconel alloy pressure vessel. This approach differs from earlier *in situ* or autogenous reactions which use Mg and amorphous boron as starting materials reacting at *T* > 650 °C. The process described in this work requires the development of autogenous pressure within the reactor initially at relatively low temperature (50 °C < *T* < 100 °C) through the decomposition of NaBH_4_ and generation of H_2_ and/or B_2_H_6_. A hold at 50 °C for 20 min enhances gas generation while, with continued heating, a hold at 250 °C for more than 20 min significantly enhances physical properties of the MgB_2_ product once heated to >420 °C.

While the detailed mechanisms that allow formation of MgB_2_ under these conditions are, as yet unclear, the overall reactions to produce MgB_2_ involve gas-solid and/or gas-liquid interaction based on known behaviour of related phases observed in reactions at lower temperatures and as minor phases in the final product. The formation of B_2_H_6_ and higher order analogues is a key step in these detailed reaction mechanisms. Data from this work suggest that higher order forms of B_2_H_6_ may also participate in higher temperature reactions (>250 °C) to form MgB_2_ and that other phases such as MgH_2_ are implicit intermediates.

The optimised product from this synthesis method forms at ~500 °C at an initial maximum autogenous pressure ~2.0 MPa after a five hour hold at 250 °C and subsequent heating at 500 °C for ten hours. This MgB_2_ forms as dense, euhedral interpenetrating forms with hexagonal habit ranging in size from 40 μm to 80 μm and a bulk T_c_ of 38.5 °C. This process to produce high quality, micron-sized MgB_2_ grains offers potential to explore growth conditions that generate high density wires, tapes or ribbons for evaluation of bulk conduction properties.

## Figures and Tables

**Table 1.** Parameters for formation of MgB_2_.

Run	*T*_max_ (°C)	Hold Temperature; Time (°C; h)	Elapsed Time *(h)	*P*_max_ (MPa)	*P*_min_ (MPa)	Major Products **
1^+^	420	50, 0.3; 250, 0.3	16	1.3	0.1	MgB_2_ (Mg, MgO)
2	500	50, 0.3; 250, 0.3	16	1.7–2.0	−0.1	MgB_2_ (MgO) NaH NaMgH_3_ (NaBH_4_)
3	500	50, 0.3; 250, 5.0	22	1.66	−0.09	MgB_2_ (NaH, MgO)
4	500	no hold	11	0.29	−0.07	MgB_2_ (MgO) NaH (NaBH_4_)

* time until reactor cooled to ~30 °C

** compounds in parentheses are in minor abundance

+ products are washed before XRD analysis, so Na products are not observed.

**Table 2.** Parameters for low temperature reactions.

Run	*T*_max_ (°C)	Hold Temperature; Time (°C; h)	Elapsed Time * (h)	*P*_max_ (MPa)	*P*_min_ (MPa)	Major Products **
5	400	50, 0.3; 250, 0.3	10	0.36	−0.04	Mg (MgB_2_) NaBH_4_ (NaH)
6	400	no hold	10	0.61	−0.02	Mg (MgB_2_) NaBH_4_ (NaH)
7	300	50, 0.3; 250, 0.3	5	0.071	−0.02	Mg MgH_2_ (MgO) NaBH_4_
8	200	50, 0.3	9	0.051	−0.03	Mg, NaBH_4_
9	200	50, 0.3	10	0.0	−0.02	Mg, NaBH_4_

* time until reactor cooled to ~30 °C

** compounds in parentheses are in minor abundance.
